# Cytoreductive surgery and hyperthermic intraperitoneal chemotherapy versus palliative systemic chemotherapy in stomach cancer patients with peritoneal dissemination, the study protocol of a multicentre randomised controlled trial (PERISCOPE II)

**DOI:** 10.1186/s12885-019-5640-2

**Published:** 2019-05-06

**Authors:** W. J. Koemans, R. T. van der Kaaij, H. Boot, T. Buffart, A. A. F. A. Veenhof, K. J. Hartemink, C. Grootscholten, P. Snaebjornsson, V. P. Retel, H. van Tinteren, S. Vanhoutvin, V. van der Noort, A. Houwink, C. Hahn, A. D. R. Huitema, M. Lahaye, M. Los, P. van den Barselaar, O. Imhof, A. Aalbers, G. M. van Dam, B. van Etten, B. P. L. Wijnhoven, M. D. P. Luyer, D. Boerma, J. W. van Sandick

**Affiliations:** 1 Department of Surgery, The Netherlands Cancer Institute-Antoni van Leeuwenhoek Hospital, Plesmanlaan 121, Amsterdam, 1066CX The Netherlands; 2 Department of Gastro-Intestinal Oncology, The Netherlands Cancer Institute-Antoni van Leeuwenhoek Hospital, Plesmanlaan 121, Amsterdam, 1066CX The Netherlands; 3 Department of Pathology, The Netherlands Cancer Institute-Antoni van Leeuwenhoek Hospital, Plesmanlaan 121, Amsterdam, 1066CX The Netherlands; 4 Department of Psychosocial Research and Epidomiology, The Netherlands Cancer Institute-Antoni van Leeuwenhoek Hospital, Plesmanlaan 121, Amsterdam, 1066CX The Netherlands; 5 Department of Biometrics, The Netherlands Cancer Institute-Antoni van Leeuwenhoek Hospital, Plesmanlaan 121, Amsterdam, 1066CX The Netherlands; 6 Department of Anaesthesiology, The Netherlands Cancer Institute-Antoni van Leeuwenhoek Hospital, Plesmanlaan 121, Amsterdam, 1066CX The Netherlands; 7 Department of Pharmacy & Pharmacology, The Netherlands Cancer Institute-Antoni van Leeuwenhoek Hospital, Plesmanlaan 121, Amsterdam, 1066CX The Netherlands; 8 Department of Radiology, The Netherlands Cancer Institute-Antoni van Leeuwenhoek Hospital, Plesmanlaan 121, 1066CX Amsterdam, The Netherlands; 9 Department of Oncology, Sint Antonius Hospital, Koekoekslaan 1, Nieuwegein, 3435 CM The Netherlands; 10 Clinical perfusion, Heartbeat, Kerkstraat 3A, Eemnes, 3755 CK The Netherlands; 11 Department of Surgery, University Medical Center Groningen, Hanzeplein 1, Groningen, 9713 GZ The Netherlands; 12 Department of Surgery, Erasmus Medical Center, Doctor Molewaterplein 40, Rotterdam, 3015 GD The Netherlands; 13 Department of Surgery, Catharina Hospital, Michelangelolaan 2, Eindhoven, 5623 EJ The Netherlands; 14 Department of Surgery, Sint Antonius Hospital, Koekoekslaan 1, Nieuwegein, 3435 CM The Netherlands

**Keywords:** Hyperthermic intraperitoneal chemotherapy, HIPEC, Peritoneal metastasis, Peritonitis carcinomatosa, Gastric cancer, Cytoreductive surgery, Palliative systemic chemotherapy, Gastrectomy, Surgery

## Abstract

**Background:**

At present, palliative systemic chemotherapy is the standard treatment in the Netherlands for gastric cancer patients with peritoneal dissemination. In contrast to lymphatic and haematogenous dissemination, peritoneal dissemination may be regarded as locoregional spread of disease. Administering cytotoxic drugs directly into the peritoneal cavity has an advantage over systemic chemotherapy since high concentrations can be delivered directly into the peritoneal cavity with limited systemic toxicity. The combination of a radical gastrectomy with cytoreductive surgery (CRS) and hyperthermic intraperitoneal chemotherapy (HIPEC) has shown promising results in patients with gastric cancer in Asia. However, the results obtained in Asian patients cannot be extrapolated to Western patients.

The aim of this study is to compare the overall survival between patients with gastric cancer with limited peritoneal dissemination and/or tumour positive peritoneal cytology treated with palliative systemic chemotherapy, and those treated with gastrectomy, CRS and HIPEC after neoadjuvant systemic chemotherapy.

**Methods:**

In this multicentre randomised controlled two-armed phase III trial, 106 patients will be randomised (1:1) between palliative systemic chemotherapy only (standard treatment) and gastrectomy, CRS and HIPEC (experimental treatment) after 3–4 cycles of systemic chemotherapy.Patients with gastric cancer are eligible for inclusion if (1) the primary cT3-cT4 gastric tumour including regional lymph nodes is considered to be resectable, (2) limited peritoneal dissemination (Peritoneal Cancer Index < 7) and/or tumour positive peritoneal cytology are confirmed by laparoscopy or laparotomy, and (3) systemic chemotherapy was given (prior to inclusion) without disease progression.

**Discussion:**

The PERISCOPE II study will determine whether gastric cancer patients with limited peritoneal dissemination and/or tumour positive peritoneal cytology treated with systemic chemotherapy, gastrectomy, CRS and HIPEC have a survival benefit over patients treated with palliative systemic chemotherapy only.

**Trial registration:**

clinicaltrials.gov NCT03348150; registration date November 2017; first enrolment November 2017; expected end date December 2022; trial status: Ongoing.

**Electronic supplementary material:**

The online version of this article (10.1186/s12885-019-5640-2) contains supplementary material, which is available to authorized users.

## Background

Gastric cancer has an aggressive natural behaviour, with 40% of the patients having metastatic disease at the time of diagnosis [1]. The peritoneum is a predilection site for tumour dissemination and is synchronously affected in 14% of all patients. Around 9% of the patients have peritoneal dissemination without other metastatic localisations. The prognosis of patients with peritoneal dissemination is dismal, with a median overall survival of only 3–4 months [1, 2].

Systemic therapy is less effective in patients with peritoneal dissemination compared to patients with metastases in other locations [3–5]. Administering cytotoxic drugs into the peritoneal cavity offers several advantages over systemic chemotherapy. Firstly, high concentrations can be delivered directly into the peritoneal cavity with limited systemic toxicity [6]. Secondly, heating enhances the cytotoxicity of some agents (e.g. cisplatin and oxaliplatin) [7, 8]. Rat models have shown enhanced tumour penetration in intraperitoneal tumour deposits if chemotherapeutic agents are administered intraperitoneally compared to intravenously [9]. Hyperthermic Intraperitoneal Chemotherapy (HIPEC) has proven its therapeutic efficacy in patients with peritoneal dissemination from several cancer types, e.g. colon cancer and ovarian cancer [10–12]. For patients with peritoneal dissemination from gastric cancer there is data, primarily Asian, suggesting that intraperitoneal chemotherapy combined with gastrectomy and cytoreductive surgery (CRS) may improve survival [13–15].

Previously, our study group conducted a phase I-II dose-escalation trial (PERISCOPE I) to study safety and feasibility of a procedure combining gastrectomy, CRS and HIPEC with oxaliplatin (41-42 °C) followed by docetaxel (37 °C) [16]. In a strictly selected group of patients, the treatment was safe and feasible with an intraperitoneal dose of 460 mg/m^2^ oxaliplatin followed by 50 mg/m^2^ docetaxel after the evolvement of a stringent post-operative care protocol [17]. In the Netherlands, the Ministry of Health appointed this novel approach as highly innovative, having led to participation in the Coverage with Evidence Development (CED) program. Within this program, the gastric HIPEC procedure is currently conditionally reimbursed by health insurance.

The primary objective of the present study is to compare overall survival between gastric cancer patients with limited peritoneal dissemination and/or tumour positive peritoneal cytology treated with the current standard treatment, i.e. palliative systemic chemotherapy, and those treated with gastrectomy, CRS and HIPEC after neoadjuvant systemic chemotherapy. Within the CED program, the second objective of this study is to calculate cost-effectiveness. If the experimental treatment provides a survival benefit over the standard treatment, health insurance coverage will be made unconditional.

## Methods

### Study design

**Fig. 1:**
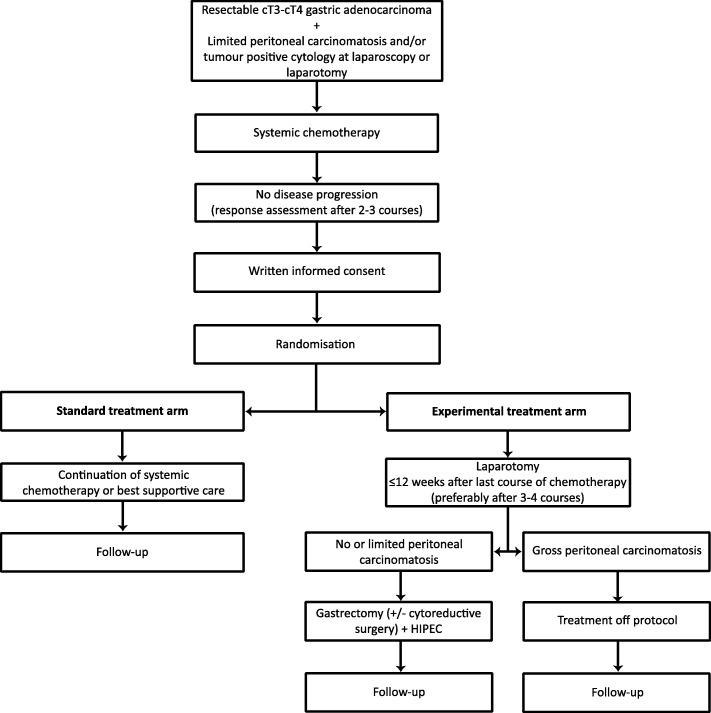
PERISCOPE II study flowchart. HIPEC: Hyperthermic Intraperitoneal Chemotherapy

The PERISCOPE II study is a multicentre randomised controlled two-armed phase III trial (Fig. 1). After 3–4 cycles of systemic chemotherapy, patients are randomly allocated (1:1) to the standard treatment arm (continuing palliative systemic chemotherapy) or to the experimental treatment arm (gastrectomy, CRS and HIPEC). The study protocol has been approved by the medical ethical committee of the Netherlands Cancer Institute-Antoni van Leeuwenhoek Hospital. A research grant has been provided by The Netherlands Organisation for Health Research and Development (ZonMW).

### Study population

Adult patients (18 years or older), with histologically proven locally advanced (cT3-cT4, any N) adenocarcinoma or undifferentiated carcinoma of the stomach with limited peritoneal dissemination and/or tumour positive peritoneal cytology are eligible for participation. In this trial, limited peritoneal dissemination is defined as a Peritoneal Cancer Index (PCI) below 7 [18–20]. At first, patients have to be treated with systemic chemotherapy. Study candidates are included provided that the primary gastric tumour is considered resectable, there is no disease progression during systemic chemotherapy and distant metastases are absent. A detailed list of the inclusion and exclusion criteria can be found in the Additional file 1.

### Sample size

Previous data have indicated that the median survival time of patients with gastric cancer with peritoneal metastasis is about 3–4 months [1]. It is expected that in the experimental arm 75% of patients will receive protocol treatment and 25% of patients will be treated off protocol due to gross peritoneal dissemination (PCI ≥ 7) at the time of the laparotomy. It is hypothesised that the median overall survival among the 75% of patients in the experimental arm who actually undergo CRS and HIPEC will be 12 months, while the other 25% of patients in the experimental arm will have a median overall survival of only 3 months.

A total of 106 patients, 53 in each arm, will be included and followed until a total of 80 deaths is observed. Assuming exponential survival with medians as described above in each of the three groups, this will yield 90% power to detect a difference in overall survival at the two-sided 95% confidence level (intention-to-treat analysis).

In the Netherlands, around 200 patients per year are diagnosed with gastric cancer and synchronous peritoneal carcinomatosis without distant metastases [1]. At least 60% of these patients will not be eligible for the study because of co-existing diseases, poor condition, irresectability of the gastric tumour and/or gross peritoneal tumour involvement. This leaves an estimated number of 80 patients per year eligible for inclusion. Next to that, around 5% of all newly diagnosed gastric cancer patients who are found suitable for treatment with curative intent has tumour positive peritoneal cytology [21, 22]. This group of patients, around 50 per year, is also eligible for inclusion. It adds up to a total of 130 potential study candidates per year in the Netherlands.

The expected accrual is around 35 patients per year. Via the Dutch Upper GI Cancer Group (DUCG) all medical oncologists and surgeons who treat patients with gastric cancer are being informed on a regularly basis about the study progress and referral issues. Patient inclusion will take about 3 years. Thereafter, there will be an additional follow-up period of 2 years, for a total study period of 5 years.

### Study procedures

### Prior to inclusion: laparoscopy or laparotomy

**Fig. 2:**
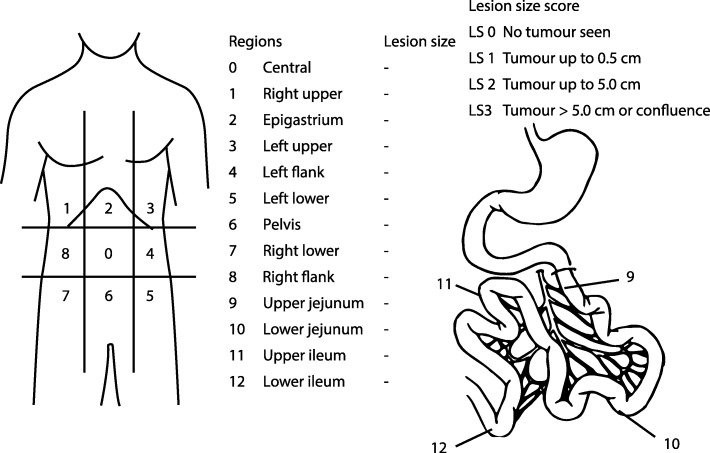
Peritoneal cancer index [18]

Prior to inclusion all patients undergo a diagnostic laparoscopy or laparotomy. During this procedure the extent of peritoneal dissemination is assessed. The presence and number of macroscopic tumour deposits are recorded according to the PCI (Fig. 2) [18].

### Prior to inclusion: systemic chemotherapy and response assessment

Patients are treated with systemic chemotherapy prior to inclusion. Accepted chemotherapy regimens generally consist of a platinum-drug combined with a fluoropyrimidine. Additionally, an anthracycline or taxane can be added according to the local protocol. Examples of accepted chemotherapy regimens are: docetaxel + oxaliplatin + 5-FU, docetaxel + cisplatin + 5-FU, epirucibin + cisplatin + 5-FU, epirucibin + oxaliplatin + 5-FU. In patients with a Her2 positive gastric tumour, trastuzumab can be added to the combination of chemotherapeutic drugs.

Response assessment is done by a Computed Tomography (CT)-scan after 2–3 courses. In the absence of disease progression, patients can be included. Response evaluation and patient’s study inclusion are discussed in (local or regional) multidisciplinary tumour board meetings.

### Inclusion and randomisation

After written informed consent is obtained, the patient is registered and randomised. Patients are randomised centrally by computer and are stratified for centre (name of hospital), main histological subtype (intestinal versus diffuse) and for the extent of peritoneal dissemination (macroscopic peritoneal tumour deposits versus tumour positive peritoneal cytology only).

### Treatment

#### Standard arm

After randomisation, patients included in the standard arm continue treatment with systemic chemotherapy. The treating physician determines which chemotherapeutic regimen is used and the duration of the treatment. Surgery in this arm is only performed to relieve severe symptoms, such as a gastric outlet obstruction.

#### Experimental arm

If allocated to the experimental treatment arm, preferably 3–4 courses of systemic chemotherapy are given prior to surgery, as is usual in the potentially curative setting for gastric cancer. Within 4 weeks before the planned operation an additional CT-scan is made. If there are still no signs of tumour progression patients proceed to surgery.

##### Laparotomy

Surgical approach is via a midline laparotomy. A thorough inspection of the peritoneal cavity is performed. If ascites is found, representative samples are obtained for cytological assessment. The presence and number of macroscopic tumour deposits are recorded to score the PCI (Fig. 2).

Gross peritoneal dissemination (PCI ≥7), small bowel dissemination and/or an irresectable primary gastric tumour preclude further study treatment. In these instances, HIPEC is not performed and it is up to the surgeon to decide whether a palliative surgical intervention is indicated.

##### Gastrectomy, cytoreductive surgery and HIPEC

If a potentially curative gastric cancer resection is possible and the PCI is below 7, a (sub)total gastrectomy with D2 lymphadenectomy is performed. Patients with macroscopic peritoneal tumour deposits undergo CRS to leave no macroscopic disease behind. Gastrointestinal continuity is restored by either a Billroth II or Roux-en-Y reconstruction.

HIPEC is performed via 3 inflow and 2 outflow catheters using an open abdominal technique under continuous circulation. The peritoneal cavity is perfused with 460 mg/m^2^ oxaliplatin (max 920 mg) at an intraperitoneal temperature of 41 °C to 42 °C. After 30 min, the perfusion fluid is drained from the abdomen and the peritoneal cavity is perfused with 50 mg/m^2^ docetaxel (max 100 mg) at an intraperitoneal temperature of 37 °C, for 90 min. A feeding jejunostomy catheter is inserted and will remain in situ until oral intake is adequate. The three inflow catheters are left in situ for postoperative drainage.

##### Postoperative care

After surgery, all patients are admitted to the Intensive Care Unit. Postoperative enteral feeding via the jejunostomy catheter can start on the day of surgery at a very low dosage (maximum 10 cc/hour). Besides that, total parenteral nutrition is started on postoperative day 3. When there are no (more) signs of a postoperative ileus, oral feeding is introduced and enteral feeding via the jejunostomy catheter is gradually increased.

##### Adjuvant treatment

Adjuvant treatment is not part of the standard study protocol but will be discussed in the multidisciplinary tumour board meeting for all patients included in the study. The decision is based upon the patient’s individual intraoperative and pathological results, the response to and toxicity from neoadjuvant systemic therapy, as well as the postoperative recovery.

### Follow-up

All patients, including those patients whose treatment has deviated from the study protocol, are seen at the outpatient clinic every 3 months for 1.5 years and every 6 months thereafter until 3 years after randomisation. Survival status and disease recurrence/progression are assessed until death. Follow-up consists of physical examination, diagnostic investigations (tumour markers in blood samples and CT-scans) and registration of hospital re-admission details (if applicable). Quality of life (QoL) questionnaires are sent to the patient at 3, 9, 15, 24 and 36 months after randomisation.

### Safety

All adverse events and serious adverse events are recorded until 100 days after randomisation (standard arm) or surgery (experimental arm). To ensure quality of data, study integrity and compliance with the protocol and the various applicable regulations and guidelines, a data monitor of the Netherlands Cancer Institute-Antoni van Leeuwenhoek Hospital has been appointed to conduct site visits to the participating centres and randomly check patient data. Data from all patients are also checked at the central data centre of the Netherlands Cancer Institute-Antoni van Leeuwenhoek Hospital. An independent safety monitoring board including a statistician, surgeon and medical oncologist has been installed. After the first 20 patients have completed 90 days of follow-up the safety monitoring board will advise on the continuation of the study. This procedure will be repeated after the inclusion of 40 patients with 90 days of follow-up.

### Analysis

Study outcome parameters will be analysed using descriptive statistical methods. Overall and disease-free survival analyses will be performed by the Kaplan-Meier method for all patients following the intention-to-treat principle. A per-protocol analysis will be performed. In these analyses, survival will be measured from the date of randomisation to the date of disease recurrence and/or death. An interim analysis for efficacy will be performed when 40 deaths (i.e. half of the required number of events) have been observed.

### Cost-effectiveness analysis

The cost-effectiveness analysis will compare the costs and health benefits of the standard treatment (palliative systemic chemotherapy) to those of the experimental treatment (including the HIPEC procedure). This analysis will include direct costs (surgery, HIPEC, diagnostic work-up, treatment of recurrences, follow-up visits and palliative care) and indirect costs such as productivity losses. The primary outcome for health effects will be quality adjusted life years, measured by means of the EuroQol 5D, being part of the study QoL questionnaires.

### Responsibilities

Protocol modifications will be submitted as amendment to the central medical ethical committee by the study coordinator. Communication between the study centres, the independent safety monitoring board and the data monitor is coordinated by the study coordinator. Participating study centres are responsible for patient inclusion, patient treatment, patient follow-up and data collection in the central data portal. At least twice a year a meeting will be organised for all relevant parties, i.e., the principle and local investigators, the trial sponsors, the data monitor, and the study coordinator, to discuss progress, problems and possible protocol modifications. The study coordinator – together with the principle investigator - will have access to the final dataset and is responsible for publishing study results. The results will be submitted to a peer-reviewed journal.

## Discussion

### Study rationale

The primary objective of the PERISCOPE II study is to compare overall survival between patients with gastric cancer with limited peritoneal dissemination and/or tumour positive peritoneal cytology treated with the current standard treatment, i.e. palliative systemic chemotherapy and those treated with gastrectomy, CRS and HIPEC, following systemic chemotherapy. In the dose-escalation PERISCOPE I study the combination of gastrectomy, CRS and HIPEC with oxaliplatin and docetaxel following systemic chemotherapy appeared safe and feasible provided that the following aspects were acknowledged [16, 17]. Firstly, the maximum dose of intraperitoneal docetaxel should not exceed 50 mg/m^2^ [17]. Secondly, patients were selected according to strict in- and exclusion criteria [16]. And, thirdly, to counteract the frequent occurrence of ileus-related postoperative complications, a stringent postoperative care protocol has been implemented [23].

### Patient selection

Complete cytoreduction is a key element in successful HIPEC surgery. There is a clear relationship between the probability to reach a complete cytoreduction and the extent of peritoneal disease, i.e. the PCI [19]. In several studies of patients with gastric cancer with peritoneal metastasis treated with a HIPEC procedure, a PCI of 7 emerged as a cut-off value between patients with long-term survival and those without [19, 20, 24]. Therefore, in the PERISCOPE II study, a PCI below 7 has been defined as inclusion criterion. It can be expected that strict PCI criteria improve homogeneity of the included study population.

### Choice of intraperitoneal chemotherapy

Based on a comprehensive literature review a combination of a platinum-based agent and a taxane was considered to be the most promising for the intraperitoneal treatment of peritoneal dissemination of gastric cancer origin [25]. Cisplatin and oxaliplatin are both platinum-based chemotherapeutic agents that are often used in HIPEC procedures. For gastric cancer patients oxaliplatin seems favourable for a number of reasons. Firstly, gastric cancer cell lines are more sensitive to oxaliplatin than to cisplatin [26]. Secondly, systemic oxaliplatin appears to be superior, or at least equal, in terms of overall and disease-free survival in patients with gastric cancer [27, 28]. And, lastly, in contrast to cisplatin, oxaliplatin is not nephrotoxic.

The taxane docetaxel was chosen as second agent as it is widely used in the systemic treatment of gastric cancer [29, 30]. It can be administered intraperitoneally, as shown in Asian studies wherein catheter-based-intraperitoneal docetaxel had clinical efficacy with acceptable safety [13, 29].

### Learning curve

HIPEC procedures in general have a steep learning curve [31–33]. In the current trial, no more than 5 centres will participate in the experimental treatment arm. These 5 centres were selected based on their experience in gastric cancer surgery and in HIPEC procedures for other indications as well as on their geographic location in the Netherlands. During the PERISCOPE I study, strict guidelines for per-operative and postoperative care were defined. Considering the extensive experience of the participating centres together with the strict guidelines, the learning curve in the PERISCOPE II is expected to be negligible.

### Cost effectiveness

Based on the costs and the quality-adjusted life years a model can be drafted to estimate the financial impact of the experimental treatment. This will provide governments with a potential basis to draft legislation regarding cost authorisation for the HIPEC procedure as a possible treatment option in the management of gastric cancer patients.

### Other HIPEC trials for gastric cancer

In the German GASTRIPEC trial (NCT02158988) gastrectomy and CRS are compared to gastrectomy, CRS and HIPEC with mitomycin C and cisplatin in patients with gastric cancer and synchronous peritoneal dissemination. In the French GASTRCHIP trial (NCT01882933) gastrectomy and HIPEC with oxaliplatin (250 mg/m^2^) are compared to gastrectomy only in patients with locally advanced gastric cancer defined as cT3-cT4 with either serosal invasion, tumour perforation, lymph node invasion or tumour positive peritoneal cytology [34]. The presence of macroscopic peritoneal lesions is an exclusion criterion in the GASTRICHIP trial. At present, the PERISCOPE II trial is unique in comparing gastrectomy, CRS and HIPEC with palliative systemic chemotherapy, which is the current standard treatment for patients with gastric cancer with peritoneal dissemination in the Netherlands.

## Conclusion

The PERISCOPE II trial will determine whether patients with gastric cancer with limited peritoneal dissemination (PCI < 7) and/or tumour positive peritoneal cytology treated with systemic chemotherapy followed by gastrectomy, CRS and HIPEC have a survival benefit over those treated with palliative systemic chemotherapy alone. The study will provide data on survival, toxicity, cost-effectiveness and quality of life in patients with gastric cancer undergoing HIPEC surgery. The ultimate goal is to define whether the HIPEC procedure can be used as a standard treatment option for patients with gastric cancer with limited peritoneal dissemination and/or tumour positive peritoneal cytology, provided that there was no disease progression during neoadjuvant systemic chemotherapy.

## Additional file

Additional file 1: Inclusion and exclusion criteria of the PERISCOPE II trial. List of all in and exclusion criteria. (DOCX 19 kb)

## Abbreviations

CED: Coverage with Evidence Development
CRS: Cytoreductive surgery
CT: Computed Tomography
HIPEC: Hyperthermic intraperitoneal chemotherapy
PCI: Peritoneal cancer index
QoL: Quality of life
TNM: Tumour Nodal metastasis
ZonMW: The Netherlands Organisation for Health Research and Development
