# Supplementary material for: Cytoreductive surgery and hyperthermic intraperitoneal chemotherapy versus palliative systemic chemotherapy in stomach cancer patients with peritoneal dissemination, the study protocol of a multicentre randomised controlled trial (PERISCOPE II)
Source: BMC Cancer. 2019 May 6;19:420. doi: 10.1186/s12885-019-5640-2 (PMC6501330; doi:10.1186/s12885-019-5640-2)
Supplement: Supplementary file 1 — Inclusion and exclusion criteria of the PERISCOPE II trial. List of all in and exclusion criteria. (DOCX 19 kb) [file 12885_2019_5640_MOESM1_ESM.docx]

**Additional file 1**

**Inclusion criteria**

In order to be eligible for participation in this study, a patient must meet all following criteria:

- Age ≥ 18 years.
- Biopsy proven primary adenocarcinoma (or undifferentiated carcinoma) of the stomach.
  Including tumours at the oesophagogastric junction provided that the bulk of the tumour is located in the stomach, and, the intended surgical treatment is a gastric resection and not an oesophagectomy. A high intra-thoracic anastomosis is allowed, but not if a thoracotomy is necessary.
- cT3-cT4 tumour (TNM classification, 7th edition) considered to be resectable (including lymph nodes).
- Limited peritoneal carcinomatosis (PCI <7) and/or tumour positive peritoneal cytology confirmed by laparoscopy or laparotomy and proven by pathological examination.
- Treatment with systemic chemotherapy, with the latest course ending within 8 weeks prior to inclusion. All currently standard chemotherapy regimens are acceptable.
- Absence of disease progression during systemic chemotherapy (prior to inclusion).
- World Health Organisation performance status 0-2.
- Adequate bone marrow, hepatic and renal function. Minimally acceptable laboratory values at start of the study inclusion:
  - White blood cell count (WBC) ≥3.0 x 10^9^ /L
  - Platelet count ≥ 100 x 10^9^ /L
  - Serum bilirubin ≤ 1.5 x ULN, and ALAT and ASAT ≤ 2.5 x ULN
  - Creatinine clearance ≥ 50 ml/min (measured or calculated by Cockcroft-Gault formula)
- For female patients who are not sterilised or in menopause (i.e., amenorrhea ≥1 year if age ≥60 years, or ≥2 years if age <60 years):
  - negative pregnancy test (urine/serum)
  - no breast feeding or active pregnancy ambition
  - reliable contraceptive methods
- Signed informed consent.

## Exclusion criteria

A patient who meets any of the following criteria will be excluded from participation in this study:

- Distant metastases (e.g., liver, lung, para-aortic lymph nodes; i.e., stations 14 and 16) or small bowel dissemination.
- Recurrent gastric cancer.
- Prior resection of the primary gastric tumour.
- Non-synchronous peritoneal carcinomatosis.
- Current other malignancy (other than cervix carcinoma and basalioma).
- Uncontrolled infectious disease or known infection with Human Immunodeficiency Virus type -1 or -2.
- A known history of hepatitis B or C with active viral replication.
- Recent myocardial infarction (< 6 months) or unstable angina.
- Any medical condition not yet specified above that is considered to interfere with study procedures, including adequate follow-up and compliance and/or would jeopardise safe treatment.
- Known hypersensitivity for any of the applied chemotherapeutic agents and/or their solvents.
